# Profiling of the Causative Bacteria in Infected Lymphocysts after Lymphadenectomy for Gynecologic Cancer by Pyrosequencing the 16S Ribosomal RNA Gene Using Next-Generation Sequencing Technology

**DOI:** 10.1155/2019/9326285

**Published:** 2019-02-21

**Authors:** Yuya Nogami, Kouji Banno, Masataka Adachi, Haruko Kunitomi, Yusuke Kobayashi, Eiichiro Tominaga, Daisuke Aoki

**Affiliations:** Department of Obstetrics and Gynecology, Keio University School of Medicine, Tokyo 160-8582, Japan

## Abstract

**Background:**

Surgery for gynecologic cancer with lymphadenectomy and pelvic radiotherapy can produce lymphoceles that sometimes complicate with infection, resulting in abscesses. The true pathogenic bacteria of abscesses are not always found because of false-negative results due to administered antibiotics and difficulty with detection, including for anaerobic bacteria. Analyzing bacteria flora by next-generation sequencing (NGS) using 16S ribosomal DNA may reveal the true pathogenic bacteria in abscesses. This is the first report on causative pathogens for infectious lymphocele using this technology.

**Methods:**

The subjects were patients who developed infectious lymphocele after surgery for gynecologic cancer at our hospital from July 2015 to September 2016. NGS analyses of bacterial flora were performed using specimens preserved at -80°C. Two steps of PCR were performed for purified DNA samples to obtain sequence libraries. Processing of sequence data, including operational taxonomic unit (OTU) definition, taxonomy assignment, and an OTU BLAST search were performed. All patients gave written informed consent and the study was approved by the institutional research ethics committee.

**Results:**

Six patients underwent puncture and drainage. The result in most cases indicated a single causative pathogen, including* Staphylococcus lugdunensis, Streptococcus dysgalactiae, Streptococcus equinus, Enterococcus saccharolyticus,* and* Escherichia coli. Conclusions*. NGS revealed that the causative bacteria in lymphocele infection are normally a single strain, such as a surface Gram-positive coccus or enteric bacteria. Antibiotics should be chosen as appropriate for elimination of these respective bacteria.

## 1. Introduction

Surgery for gynecologic cancer with lymphadenectomy and pelvic radiotherapy can induce lymphatic congestion and produce lymphoceles. Lymphoceles themselves are not a clinical problem, but sometimes complicate with infection, resulting in abscesses. Patients who are oncologically cured but develop these problems may have reduced quality of life, and these episodes can occur immediately to more than 10 years after surgery [1].

Infectious lymphoceles that develop later after surgery are particularly considered to be caused by bacteria invading through a wound in the lower extremity and perineum, similarly to cellulitis; however, most such lymphocele have no identified trigger. Therefore, an infectious lymphocele is thought to have an etiology that differs from that of pelvic abscess derived from the gastrointestinal tract, with involvement of different pathogenic bacteria [2, 3]. Antimicrobial treatment and drainage are recommended, but the abscesses are present in the deep pelvis. Relatively safe procedures including computed tomography (CT)-guided drainage have been established, but a risk for complications still remains. Therefore, the indication should be carefully selected. Conservative therapy with antibiotics is often used initially, and then a puncture is taken when a patient does not recover [4, 5].

For patients who are improved by conservative therapy, the pathogenic bacteria are often unknown. Furthermore, fluids collected from an abscess are usually examined by Gram staining, culture tests, and sensitivity analysis; however, the true pathogenic bacteria are not always found because of false-negative results due to administered antibiotics and difficulty with detection, including for anaerobic bacteria. Identification rates by regular culture tests in previous case reports have ranged from 46% to 100% [2, 4, 6].

Recently developed next-generation sequencing (NGS) can now be used to detect true pathogenic bacteria in abscesses [7, 8]. This is the same methodology as that used in studies of the microbiome. In NGS, bacterial flora are analyzed using 16S ribosomal DNA. Dead bacteria can also be detected, which means that bacteria are detectable after the start of antibiotic treatment, and true pathogenic bacteria in abscesses can be detected even if they are less sensitive in a normal culture test. Here, we provide the first report of infectious lymphocele after treatment of gynecologic cancer in which the pathogenic bacteria were detected by NGS.

## 2. Materials and Methods

### 2.1. Patients

The subjects were patients who were treated for infectious lymphocele after surgery for gynecologic cancer at our hospital from July 2015 to September 2016. Infectious lymphocele was diagnosed on the basis of fever, local spontaneous pain, tenderness, inflammatory findings in blood test data, and inflammation based on enhanced imaging effects in surrounding regions. Patients were treated with antibiotics only and then underwent percutaneous drainage if no improvement occurred. Improvement was judged by attending doctors based on alleviation of pain, fever, and inflammatory findings in blood test data. Drained samples were used in a regular culture test and part of each sample was immediately stored at -80°C. Patients were clinically treated based on the results of the regular culture tests and all infections were remitted. The samples stored at -80°C were submitted for NGS analyses. Consequently, treatment did not depend on data from these samples. All patients gave written informed consent after receiving an oral explanation of the study. This study was approved by the institutional research ethics committee (approval no. 20140406) and is registered in the UMIN Clinical Trials Registry (http://www.umin.ac.jp/ctr/index-j.htm).

### 2.2. Next-Generation Sequencing

Purulent drainage samples were stored at -80°C temperature until DNA extraction. Genomic DNA was isolated using a NucleoSpin Microbial DNA Kit (Macherey-Nagel, Düren, Germany). The extraction protocol was performed according to the manufacturer's instructions. DNA samples were extracted from approximately 500 *µ*l of purulent contents of each abscess, but the DNA concentration of the sample from case 4 was too low for subsequent analysis. Thus, extraction was performed again using all the remaining samples from case 4.

### 2.3. Sequencing of the 16S rRNA Gene

Two steps of PCR were performed for the purified DNA samples to obtain sequence libraries. The first PCR was performed for amplification using a 16S (V3-V4) Metagenomic Library Construction Kit for NGS (Takara Bio Inc., Kusatsu, Japan) with primer pair 341F (5′-TCG GCA GCG TCA GAT GTG TAT AAG AGA CAG CCT ACG GGN GGC WGC AG-3′) corresponding to the V3-V4 region of the 16S rRNA gene. The second PCR was performed to add the index sequences for the Illumina sequencer with a barcode sequence using the Nextera XT Index Kit (Illumina, San Diego, CA). The prepared libraries were subjected to sequencing of 250 paired-end bases using the MiSeq Reagent Kit v.3 on the MiSeq (Illumina) at Takara Bio. Processing of sequence data, including operational taxonomic unit (OTU) definition, taxonomy assignment, and an OTU BLAST search were performed using CD-HIT-OTU 0.0.1, QIIME ver. 1.8, and the DDBJ 16S rRNA database, respectively.

## 3. Results

Nine patients were treated for infectious lymphocele during the study period. Three of these patients were improved by conservative therapy with antibiotics alone, and six patients underwent puncture and drainage. Blood culture was collected from all patients and a regular culture test was performed in patients who underwent drainage. Age, underlying disease, surgical history, time from surgery, immunosuppression including chemotherapy, use of antibiotics before drainage, time until drainage, abscess size on MRI, status of aspirates, blood culture results at onset, and results of NGS of bacterial flora are shown in Table 1. In the 6 cases, development of abscess ranged from immediately after surgery to more than 10 years after surgery. Two subjects underwent chemotherapy and developed abscess during the recovery phase. No patient had neutropenia of grade 3 or higher. Only one subject was positive in blood culture. Most aspirates were yellow and clear, and few showed apparent purulence. Clear yellow fluids are frequently found in such an abscess. Diagnosis of infectious lymphocele was made based on fever, local spontaneous pain, tenderness, inflammatory findings on imaging, and rapid improvement of symptoms by drainage, even if there was no purulent content.

A homology search was performed using BLASTN with a representative sequence of the OTU as a query in the DDBJ 16S rRNA database (obtained March 6, 2018). Top hits were identified as bacteria names. The results are shown in Table 2. 16S DNA of a small-scale relevant bacterium is amplified by PCR, and this allows environmental and contaminating bacteria to be identified. After eliminating frequently identified bacteria in the environment and contaminants, 5 of 6 cases were found to be due to single infection by* Staphylococcus lugdunensis, Enterococcus saccharolyticus, Escherichia coli, Streptococcus dysgalactiae, *and* Streptococcus equinus*. These results showing many cases of infection by a single strain such as a surface Gram-positive coccus or enteric bacteria were similar to those in previous study of pathogenic bacteria detected using regular culture tests [2]. The analysis using NGS did not find hidden pathogenic bacteria; therefore, double infection including anaerobic bacteria rarely occurs in infectious lymphocele after surgery for gynecological cancer.

## 4. Discussion

In this study, drainage was not routinely performed and the indication was judged from clinical findings by attending doctors. Therefore, preemptive administration of antibiotics was conducted in all subjects, and puncture was performed due to difficulties with control of infection in 6 of the 9 subjects. In regular culture tests, half the subjects were negative due to the effect of the preemptive antibiotics. In contrast, bacteria were detected in NGS in all cases. Blood culture collected from all subjects at onset was only positive in one case, which suggests difficulties in detection of pathogenic bacteria in blood culture for this infectious disease.

This study was conducted to identify the true pathogenic bacteria, and NGS was performed retrospectively after all samples were collected from patients. The results of this study will provide useful information to select antibiotics in empiric therapy for infected lymphoceles. Furthermore, the high detection rate of causative agents suggests the utility of NGS. At present, it is still expensive to detect bacteria for each patient using NGS, but this procedure promptly provides correct results that can be used for clinical diagnosis during treatment [8]. In the future, reduction of the costs and time for NGS may allow clinical judgments using this approach. However, a regular culture test is also necessary to determine antimicrobial susceptibility.

All samples in the study were collected by drainage after treatment with antibiotics. NGS can identify dead bacteria if 16S rDNA is detected, and the sensitivity of NGS is far superior to that of a regular culture test. However, the possibility of false-negative findings due to treatment cannot be ruled out because the number of reads was decreased by treatment [8].

The results indicated that complex infection with multiple pathogens, including anaerobic bacteria, seems to be rare. However, multiple bacteria were detected by NGS in one case. Case 6 had a lymphocele that developed immediately after surgery that included intestinal resection. Cysts of large sizes developed on both sides. The bacterial strain in case 6 was often detected in the environment and may be a contaminant. However, lymphocele is theoretically continuous with the intestine. In these cases, it is important to keep complex infection in mind in choosing antibiotics for empiric therapy and determining the duration of administration. Actually, in case 6, no pathogenic bacteria were detected in a regular culture test; however, long-term administration of oral levofloxacin was required due to symptom flare-up, including fever.

In case 4,* Streptococcus agalactiae* was found in a regular culture test, but* Streptococcus dysgalactiae* was detected in NGS. Prolex (Pro-Lab Diagnostics, Richmond Hill, ON, Canada) was used to detect B antigen in a latex agglutination test using Group B Streptococcus.* Streptococcus dysgalactiae* accounted for the top 5 strains based on concordance rates in NGS, which suggests failure in regular culture tests.* Acinetobacter johnsonii* was also found in many cases in this analysis, which may have been due to contamination during puncture, storage, transport, and analysis.

## 5. Conclusion

True pathogenic bacteria in infectious lymphoceles were analyzed using NGS. As in previous studies using conventional culture tests, NGS showed that the causative bacteria in lymphocele infection are a single strain, such as a surface Gram-positive coccus or enteric bacteria. Antibiotics should be chosen appropriately for elimination of these respective bacteria.

## Figures and Tables

**Table 1:** Summary of the six cases in the study.

Case Number	Postoperative period	Immuno suppression	Blood culture	Treatment before drainage		Size on MRI (width/length/height)	Culture results	Properties of abscess sample
				antibiotics	days	(mm)		
1	10 Y 9 M	None	Negative	CPFX(OA)	1	26*∗*29*∗*67	Negative	Light yellow transparent
				CZOP	4			

2	9 Y	None	Negative	CZOP	8	57*∗*32*∗*66	Negative	Light yellow transparent

3	3 M	During CTx	*Escherichia coli*	CZOP	6	61*∗*51*∗*100	*Escherichia coli*	Yellow purulent

4	7 M	During CTx	Negative	CFPM	2	88*∗*57*∗*87	*Streptococcus agalactiae*	Light yellow transparent

5	6 M	None	Negative	CFPX(OA)	14	30*∗*52*∗*42	*Streptococcus equinus*	Transparent
				CVA/AMPC	1		*Streptococcus bovis* group	

6	10 days	Postoperative	Negative	CZOP	1	lt 78*∗*120*∗*85	Negative	Light yellow transparent
				PIP/TAZ	7	rt 110*∗*76*∗*210	Negative	Light yellow transparent

CTx: chemotherapy, CPFX(OA): ciprofloxacin(oral administration), CZOP: cefozopran, CFPM: cefepime,

CVA/AMPC: potassium clavulanate/amoxicillin, PIP/TAZ: piperacillin/tazobactam.

**Table 2:** Results of analysis of bacteria flora using NGS.

Case Number							
1	*Staphylococcus lugdunensis*	*Acinetobacter johnsonii*					
2	*Enterococcus saccharolyticus*	*Acinetobacter johnsonii*					
3	*Escherichia coli*						
4	*Streptococcus dysgalactiae*	*Acinetobacter johnsonii*					
5	*Streptococcus equinus*						
6 rt	*Pseudomonas pseudoalcaligenes*	*Acinetobacter johnsonii*	*Propionispora* sp.				
6 lt	*Pseudomonas pseudoalcaligenes*	*Acinetobacter johnsonii*	*Pseudomonas fluorescens*	*Bradyrhizobium *genosp. O	*Pseudomonas fluorescens*	*Novosphingobium aquaticum*	*Sphingomonas* sp.
						Glaeser et al. 2013	

## Data Availability

The NGS results data used to support the findings of this study are included within the article.
